# Low-Cost Image Compressive Sensing with Multiple Measurement Rates for Object Detection

**DOI:** 10.3390/s19092079

**Published:** 2019-05-05

**Authors:** Longlong Liao, Kenli Li, Canqun Yang, Jie Liu

**Affiliations:** 1 College of Computer, National University of Defense Technology, Changsha 410073, China; 2 College of Computer Science and Electronic Engineering, Hunan University, Changsha 410082, China; lkl@hnu.edu.cn; 3 College of Computer, National University of Defense Technology, Changsha 410073, China; canqun@nudt.edu.cn (C.Y.); liujie@nudt.edu.cn (J.L.)

**Keywords:** compressive sensing, multiple measurement rates, object detection, compression ratio, half-precision float

## Abstract

When measurement rates grow, most Compressive Sensing (CS) methods suffer from an increase in overheads of transmission and storage of CS measurements, while reconstruction quality degrades appreciably when measurement rates reduce. To solve these problems in real scenarios such as large-scale distributed surveillance systems, we propose a low-cost image CS approach called MRCS for object detection. It predicts key objects using the proposed MYOLO3 detector, and then samples the regions of the key objects as well as other regions using multiple measurement rates to reduce the size of sampled CS measurements. It also stores and transmits half-precision CS measurements to further reduce the required transmission bandwidth and storage space. Comprehensive evaluations demonstrate that MYOLO3 is a smaller and improved object detector for resource-limited hardware devices such as surveillance cameras and aerial drones. They also suggest that MRCS significantly reduces the required transmission bandwidth and storage space by declining the size of CS measurements, e.g., mean Compression Ratios (mCR) achieves 1.43–22.92 on the VOC-pbc dataset. Notably, MRCS further reduces the size of CS measurements by half-precision representations. Subsequently, the required transmission bandwidth and storage space are reduced by one half as compared to the counterparts represented with single-precision floats. Moreover, it also substantially enhances the usability of object detection on reconstructed images with half-precision CS measurements and multiple measurement rates as compared to its counterpart, using a single low measurement rate.

## 1. Introduction

### 1.1. Challenges and Motivations

High-resolution cameras and drones are increasingly used for capturing images in large-scale distributed surveillance systems. The objective is to ensure the security and safety of materials, people, and places in the real world, such as smart cities, retail stores, and public transportation [1,2]. Massive amounts of images and videos collected over large-scale surveillance networks make the timely transmission of all captured data to remote servers as well as permanent storage an impractical one. Even when servers provide adequate storage space, greater consideration must be assigned to the cost of high-speed network transmission as well as increasing storage space. Moreover, the emerging trend involves real-time analysis of captured surveillance data (e.g., object detection) on intelligent surveillance devices using deep learning methods [3]. Thus, for large-scale distributed surveillance systems, it is a great challenge to reduce the required transmission bandwidth and storage space while preserving the usability of sampled images/videos.

Compressive Sensing (CS) theory demonstrates that images and videos can be reconstructed with a high probability when there is sparsity in certain transformed domains [4,5]. Meanwhile, CS can perform data sampling and compression simultaneously. It is suitable for use in limited transmission bandwidth and storage space. CS enables the low-cost acquisition of images/videos from a variety of acquisition devices such as surveillance cameras and aerial drones [6]. The CS method is especially fit for resource-constrained image capturing devices such as aerial drones and robots since it has the potential to significantly reduce the cost of image/video transmission and storage. However, most existing CS algorithms use a single measurement rate to sample and reconstruct different images [7]. They are limited to improve the performance of CS methods for an entire image, such that the reconstruction quality is substantially degraded as measurement rate reduces. By contrast, the size of measurement data increases rapidly as measurement rate grows. Thus, it is very challenging for CS sampling to use as few measurements as possible while recovering images with adequate local features that users are interested in.

Therefore, we propose a low-cost image CS approach called MRCS, which employs multiple Measurement Rates (MRs) for object detection applications in real scenarios. MRCS aims to reduce the sample data’s overheads of network transmission and storage space. It also aspires to preserve adequate image features of key regions in object detection-based scenarios such as ’Who is the person in the surveillance image?’. MRCS comprises of three stages: object detection, CS sampling with multiple MRs on terminal devices, and CS reconstruction on remote servers. To accelerate object detection on resource-constrained surveillance devices while improving the accuracy of object detection, we propose a smaller object detector architecture named MYOLO3. It is a one-stage objector that achieves the trade-off between the accuracy and number of operations measured by multiply-adds. Then, for an image/video frame, the key regions that users are interested in are sampled with higher MRs to preserve image features, while other regions are sampled with lower MRs in order to reduce the amount of measurement data. Finally, the natural images for object detection are reconstructed using CS measurements sampled with multiple MRs, which is employed as a post-acquisition step on high-performance servers [8].

### 1.2. Contributions

We mainly focus on presenting a low-cost image CS method with multiple MRs for applications related to object detection. However, our proposed method can be readily extended to the CS sampling and reconstruction of videos. The main contributions of our paper are summarized as follows.

- To reduce the model size and computation cost while improving the accuracy of object detection in MRCS, we propose a smaller real-time object detector with a depthwise feature pyramid network name MYOLO3. MYOLO3 is mainly built on bottleneck residual blocks and depthwise separable convolutions, instead of standard residual blocks and convolutions.
- To reduce the required transmission bandwidth and storage space for CS measurements, we propose a CS approach with multiple MRs for sampling natural images known as MRCS. MRCS uses higher MRs on image regions that users are interested in, while adopting lower MRs on other image regions to sample an entire image.
- We propose a half-precision presentation method of CS measurements to further reduce the size of CS measurements, which represents the values of CS measurements with 16-bit half-precision floats instead of 32-bit single-precision floats.

## 2. Related Work

### 2.1. Object Detection

Object detection is a fundamental problem in computer vision [9,10,11,12]. Driven by various Convolutional Neural Networks (CNNs), there are two different approaches to perform object detection: two-stage detectors and one-stage detectors [13,14,15]. Inspired by the success of the R-CNN [16] architecture, the two-stage object detection networks, such as Fast R-CNN [17], Faster R-CNN [18], R-FCN [19], Feature Pyramid Networks (FPN) [20] and Cascade R-CNN [21], are proposed in recent years. Meanwhile, one-stage object detection networks such as SSD [22], YOLOv2 [23] and YOLOv3 [24] become popular due to their computational efficiency by performing predictions of locations and classes in one network simultaneously.

Recently, real-time object detectors using a small network have attracted much attention from the field of deep learning based on pure CPUs and mobile devices [25]. For instance, the smaller version of YOLOv2 and YOLOv3, i.e., Tiny-YOLOv2 [23] and Tiny-YOLOv3 [24], has fewer layers and parameters than the corresponding ones of the full version. They are accelerated with smaller network architecture, but leading to the loss in the accuracy. MobileNet [26] uses depthwise separable convolutions to build a simple and homogeneous network architecture. It achieves superior performance while reducing the computations and the number of parameters of the network [27,28]. The MobileNet-based object detector MobileNet-SSD [26] achieves comparable results to other networks with only a fraction of computational complexity and model size. To design a tiny network for reducing the number of parameters while persevering the accuracy, the newly updated network MobileNetV2 introduces an inverted residual with the linear bottleneck [29]. However, real-time object detection in real-world applications still faces challenges from model size, computation cost, and accuracy.

### 2.2. CS Construction

The existing CS methods are mainly classified into two categories: iterative optimization-based CS methods and neural network-based CS methods.

Dong et al. [30] propose a Nonlocal Low-rank Regularization (NLR) for applying structured sparsity to recovery CS images. Fei et al. [31] present a CS method based on iterative directional total variation refinement. Metzler et al. [32,33] employ the unrolled optimization technique to improve the performance of the D-AMP algorithm. Majumdar and Ward [34] apply CS techniques in the reconstruction of color images. These iterative optimization-based CS methods often have theoretical convergence guarantees, but cannot fully use potentially available training data.

Inspired by the powerful learning ability of Deep Neural Networks (DNNs), several DNN-based CS approaches have been proposed. Mousavi et al. [35] use Stacked Denoising Auto-encoders (SDA) to recover images from CS measurements. DeepInverse [36] uses pure convolutional layers to build the DNN model for image CS reconstruction. ReconNet [37] uses fully connected layers and convolutional layers to create the DNN model for regressing image blocks from its CS measurement. ISTA-Net [38] casts the Iterative Shrinkage-Thresholding Algorithm (ISTA) into a DNN, and applies an effective strategy to solve proximal mapping associated with the sparsity-inducing regularizer. Yao et al. [39] present a deep residual reconstruction network for image CS, which consists of fully connected layers and residual learning blocks.

These DNN-based methods are data-driven methods that use no hand-designed models. They dramatically reduce time complexity as compared to iterative optimization-based reconstruction methods. Unfortunately, the existing methods must be trained for certain specific measurement rates, and then only perform CS sampling and reconstruction with one single MR.

## 3. Overview of Proposed MRCS

**Figure 1:**
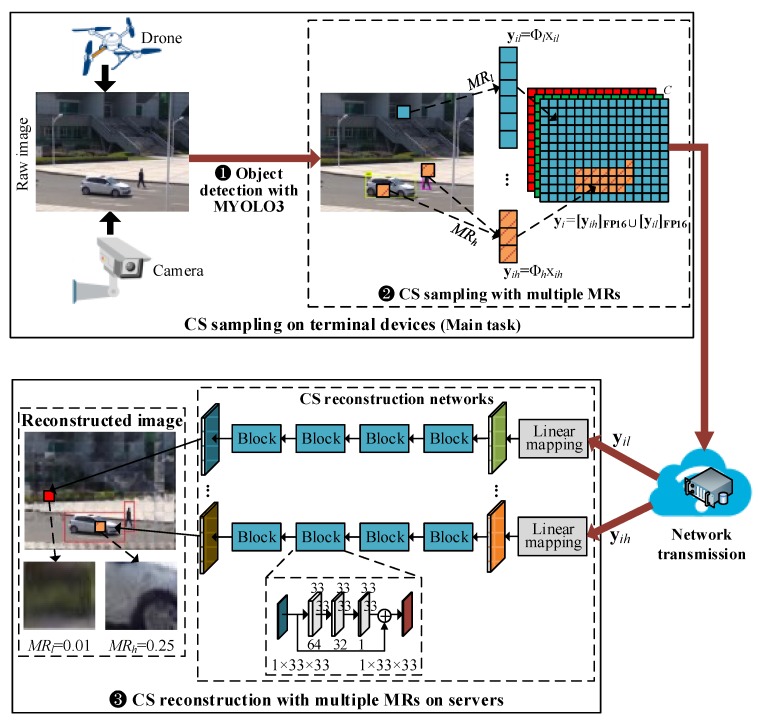
Illustration of the proposed MRCS. The main task of MRCS is CS sampling of natural images with multiple Measurement Rates (MRs), e.g., 0.25 and 0.01. [*]FP16 denotes CS measurements are represented with half-precision floats.

To preserve the complex texture of regions of interest and to reduce the cost of network transmission and storage of images capturing from large-scale surveillance cameras/systems, we propose a low-cost image CS approach with multiple measurement rates for object detection named MRCS. Figure 1 presents a case of MRCS that uses two MRs, but can readily be extended for use in cases with more than two MRs. MRCS consists of two modules: CS sampling with multiple MRs as a pre-acquisition procedure on terminal devices such as surveillance cameras and aerial drones, and CS reconstruction with corresponding multiple MRs as a post-acquisition procedure on high-performance computers or cloud servers. The main task of MRCS is to process the first module that comprises two stages, i.e., ❶ real-time object detection using the proposed MYOLO3 on captured raw images/video frames, and ❷ CS sampling of the images/video frames with multiple MRs to generate half-precision CS measurements. After the CS measurements are transmitted to remote servers by communication networks, the second module of MRCS performs the final processing stage, i.e., ❸ The received CS measurements are inputted to the corresponding CS reconstruction networks for recovering natural images with multiple MRs.

The architecture of the proposed real-time object detector MYOLO3 is illustrated in detail in Section 4, while the procedures of the second and third stage of MRCS is discussed in Section 5.

## 4. Architecture of MYOLOv3

**Figure 2:**
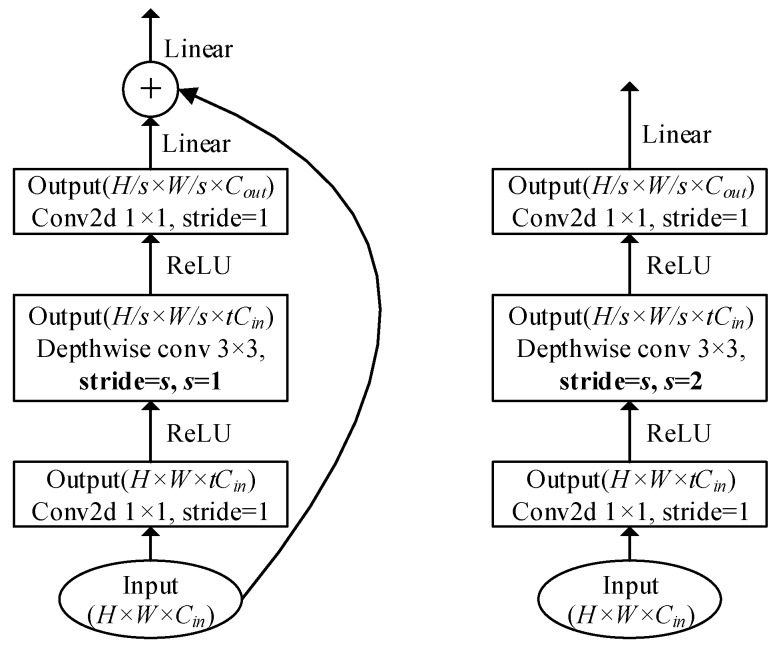
Structure of bottleneck convolution blocks transforming from $C_{in}$ input channel to $C_{out}$ output channels, with an expansion factor *t*. Left: a block for the depthwise convolution with stride = 1. Right: a block for the depthwise convolution with stride = 2.

**Figure 3:**
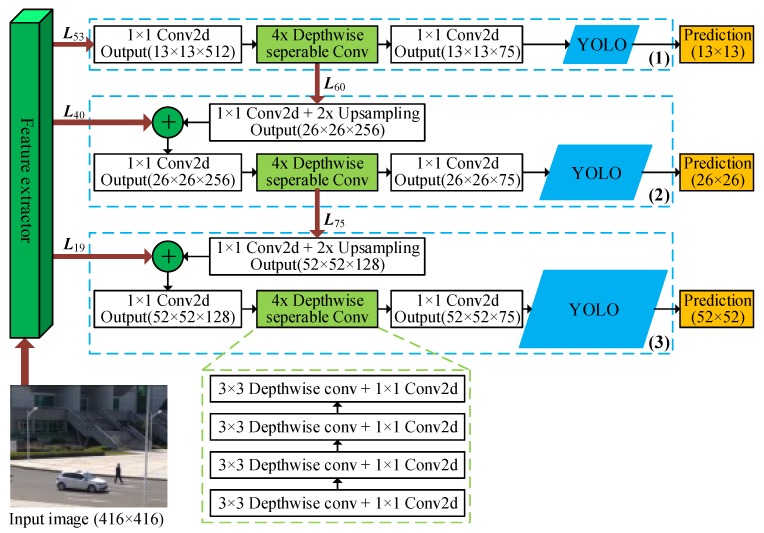
Illustration of the proposed depthwise feature pyramid network. ⨁ denotes a route layer.

The architecture of the proposed real-time object detection network MYOLO3 for image CS with multiple MRs is defined in Table 1. It contains two components: a feature extractor (i.e., the layers indexed 1–53) and a detector (i.e., the layers indexed 54–94). The feature extractor starts with an initial standard convolutional layer with 32 filters of size 3×3, following 17 bottleneck residual blocks as indicated in Figure 2 and a standard convolutional layer with 1280 filters of size 1×1. To improve the accuracy of object detection, the detector is implemented with the proposed depthwise feature pyramid network as shown in Figure 3.

The difference from YOLOv2 [23] and YOLOv3 [24], the MYOLO3 is mainly built on bottleneck residual blocks and depthwise separable convolutions, instead of standard residual blocks and conventional convolutions. Moreover, a bottleneck residual block-based MobileNetv2 is used to extract feature maps, and then a newly designed depthwise feature pyramid network is used to predict the locations and scores of detection objects. There are three reasons for this: (1) Compared with standard residual blocks, bottleneck residual blocks enable created networks to obtain improved accuracy by outputting linear activations, and have less computation cost. (2) Depthwise separable convolutions have fewer parameters than standard convolutions, and thus are less prone to over-fitting. (3) With fewer parameters, depthwise separable convolutions also require fewer computations and thus are faster.

In the following subsections, the components of MYOLO3, such as depthwise separable convolutions, bottleneck residual blocks and the designed depthwise feature pyramid network, are discussed in detail.

### 4.1. Depthwise Separable Convolutions

The depthwise separable convolution factorizes a standard convolution into a depthwise convolution for filtering and a pointwise convolution for combining [27]. The depthwise convolution is a per channel convolution that applies a single filter to each input channel. After splitting the input feature maps and filtering into channels, it convolves the input feature maps with the corresponding filters along each channel, and produces an output feature map for each channel. The pointwise convolution is a convolution with filters of size 1 × 1 to combine depthwise convolution outputs across the output channels [26].

This factorization drastically reduces the computation cost and model size. Compared with a standard convolution that takes a $H\times W\times C_{in}$ feature map as input, the filter size is K×K, the number of output channels is $C_{out}$, and the stride is 1, the depthwise convolution reduces the number of parameters by a factor of $1/C_{out}+1/K^{2}$, and also reduces computation cost by the factor of $1/C_{out}+1/K^{2}$. MYOLO3 uses 3×3 depthwise separable convolutions, resulting in the reduction of computation cost and the number of parameters by almost 9 times smaller than that of standard convolutions.

### 4.2. Bottleneck Residual Blocks

Figure 2 illustrates the structures of bottleneck residual blocks, which incorporate inverted residuals and linear bottlenecks. Each block uses a three-layer stack. The first layer is an expansion layer that implements a non-linear transformation of the input feature maps, including a 1×1 pointwise convolutional layer with stride = 1 and an activation function ReLU. The second layer is a 3×3 depthwise convolutional layer with stride = *s* and an activation function ReLU. The last layer is a linear bottleneck layer consisting of a 1×1 pointwise convolution with stride = 1 and a linear activation function. The expansion factor *t* is used to resize the input feature maps, wherein most expansion factors are set as 6 in the proposed MYOLO3. During training, batch normalization is also used behind each layer.

As illustrated in the left side of Figure 2, when the stride is 2 for the depthwise convolution, a shortcut connection is used to connect the beginning and end of the bottleneck residual block. The linear activation function is used in the last layer and after the addition operation instead of the ReLU function, since the linear transformation prevents the ReLU transformation from destroying too much information [40].

Compared with the depthwise separable convolution, the bottleneck residual block shown in Figure 2 has an extra computation cost $C_{in}\times tC_{in}\times H\times W$ from the expansion layer. However, it enables the processing of much smaller input and output feature maps. Moreover, compared with a standard residual block, the bottleneck residual block has reductions in the number of parameters of $tC_{in}\times K^{2}\times \left(tC_{in}-1\right)$, as well as in the computation cost of $tC_{in}\times H\times W\times K^{2}\times \left(tC_{in}-1\right)$.

### 4.3. Depthwise Feature Pyramid Network

As shown in Figure 3, the depthwise feature pyramid network consists of three sub-detectors. Each sub-detector begins with a standard convolutional layer with 1×1 filters, following four depthwise separable convolutions, a standard convolution with 1×1 filters and a YOLO layer. The YOLO layer performs detection predictions (e.g., coordinates of bounding boxes and class labels) [24]. The remaining layers (e.g., upsampling layers and route layers) are responsible for upsampling and concatenating feature maps to produce the input feature maps for the latter two sub-detectors. The number of filters used in the last convolutional layer (e.g., the 63rd, 69th and 93rd layer) of each sub-detector is determined by the number of detected object classes, which is denoted by the parameter cls in Table 1. As the following experiments are executed on the VOC dataset containing 20 classes (e.g., cls=20). Thus, the number of the filters in three YOLO layers is obtained as (20+5)×3=75, which is identical with the number of output channels for these convolutional layers.

Three sub-detectors make up a convolutional network with a top-down pathway and lateral connections, so that it efficiently constructs a rich, multi-scale feature pyramid from a single resolution input image. The sub-detectors conduct predictions at three different scales (i.e., 32, 16, and 8) respectively. To be more specific, the input image is divided into 32×32, 16×16, and 8×8 grid cells. With an input image of size 416×416, the grid cells produced are 416/32×416/32=13×13=169, 416/16×416/16=26×26=676 and 416/8×416/8=52×52=3704 respectively. The first sub-detector performs predictions using 13×13 feature maps. Next, the second sub-detector predicts by using the 26×26 output feature maps. A similarity process is performed to produce 52×52 input feature maps for predictions of the third sub-detector. In the end, YOLO layers output the final detected objects.

#### 4.3.1. Nearest Neighbor Upsampling Layers

To detect small objects by increasing spatial resolution, upsampling layers are used before the convolutions in the second and third sub-detectors. The upsampling layer upsamples the output feature map of the previous layer by a factor of a factor of stride, which is equivalent to 2 in the proposed MYOLO3. During forward propagation, the upsampling layer resizes the input feature maps using input stride *s*. Each 1=1×1 pixel is scaled into each $s^{2}=s\times s$ pixels, and each newly created $s^{2}-1$ pixels are filled with the value of the 1=1×1 pixel. Thus, an input $W\times H\times C_{in}$ feature map is scaled into an output $\left[W\times s\right]\times \left[H\times s\right]\times C_{in}$ feature map. In contrast, it sums values from each $s^{2}=s\times s$ pixels to a 1=1×1 pixel during backward propagation, i.e., it resizes a $W\times H\times C_{in}$ feature map to $\left[W/s\right]\times \left[H/s\right]\times C_{in}$. Compared to other upsampling methods (e.g., bilinear one), the upsampling implemented with the nearest neighbor algorithm has less computational cost since it involves no multiplication operations.

#### 4.3.2. Route Layers

The route layer is a residual-like utility layer that takes a feature map from a previous layer and merges it with an upsampled feature map using concatenation [20]. This method enables the depthwise feature pyramid network to obtain more meaningful semantic information from the upsampled features, and acquires more fine-grained information from the previous feature maps. When a route layer only takes the result of a preceding layer as input, it output the input feature map without any processing. When a route layer takes the results of more than just one preceding layer as its input, it merges the input feature maps into a single output feature map along the channel dimension using concatenation.

#### 4.3.3. YOLO Layers

For a 416×416 input image, the depthwise feature pyramid network generates a total of (13×13+26×26+52×52)×3=10647 output predictions. Each prediction contains center coordinates, dimensions (height and width), objectness score, and class confidences of predicted bounding boxes. To obtain accurate predictions, the YOLO layer performs as follows. First, it filters most of the bounding boxes based on their object scores using a defined confidence threshold (e.g., 0.5). The bounding boxes with object scores below the confidence threshold are ignored to only output bounding boxes for objects that are likely to be in the image. Second, since multiple high-confidence predictions may describe the same object, it removes redundant bounding boxes using Non-maximum Suppression (NMS) [41] to output only a single predicted bounding box for describing each detected object.

## 5. Compressive Sensing with Multiple MRs

### 5.1. CS Sampling with Multiple MRs

The CS sampling of a natural image consists of three independent CS processes on *c* channels (e.g., R, G and B), i.e., $y_{i}=\Phi x_{i},i\in 1,2,...,c$, where $y_{i}\in R^{M}$ denotes the randomized CS measurement values on the *i*-th channel, $\Phi \in R^{M\times N}$ is the measurement matrix per block for each channel, $x_{i}\in R^{N}$ denotes the original image on the channel *i*. As M≤N, the measurement rate is defined as $\frac{M}{N}$.

In addition, compared to the 32-bit single-precision float, the 16-bit half-precision float (FP16) has a limited numerical range, and consists of 1 sign bit, 5 bits of exponent, and 10 fractional bits [42]. Research has shown that there is only minor degradation in prediction accuracy when the 32-bit single float-pointing parameters of DNN models are replaced with the FP16 ones [43]. For transmission purposes, CS measurements are often not only compressed, but also quantized [44]. Merve et al. use quantization functions to quantize CS measurements, and the robustness results prove that the mean recovery error can be controlled by the quantization error [45]. Cerone et al. present a linear programming approach to quantize CS measurements and represent them in low-precision [46]. Therefore, MRCS uses FP16 to represent CS measurements for the reduction in the transmission bandwidth as well as the required storage space.

Assuming that *W* and *H* denote the width and height of the input image, $p_{st}=1$ denotes the image patch $p_{st}$ and one of the detected bounding boxes contains the same pixels, while $p_{st}=0$ denotes the opposite case. To reduce improve the compressive ratio and reduce required transmission bandwidth of CS measurements while preserving the performance of CS reconstruction, regions containing objects are sampled with higher MRs, but the remaining regions are sampled with a lower measurement rate. Thus, the CS sampling with multiple MRs in MRCS is formulated as Algorithm 1.

**Algorithm 1** The algorithm for CS sampling with multiple MRs in the proposed MRCS**Input:** A natural image of W×H with c(c≥1) channels, and *k* measurement matrices such as $\Phi_{h}$ generated with a higher measurement rate and $\Phi_{l}$ generated with a lower measurement rate.**Output:** The results of half-precision CS measurement y
1:Detect the bounding boxes of *n* target objects (e.g., persons, bicycles and cars) with the proposed object detector MYOLO3. The bounding box $b_{j}\left(j<n\right)$ is denoted as $\left[x_{j},y_{j},w_{j},h_{j}\right]$, where $x_{j}$ and $y_{j}$ declare the coordinates of its top-left corner, $w_{j}$ and $h_{j}$ declare its width and height respectively.2:Divide the input image into patches of size 33×33 without overlap, and get wp×hp=ceil((W−1)/33+1)×ceil((H−1)/33+1) image patches.3:Declare a wp×hp matrix $P=\left\{p_{st}\right\},p_{st}\in \left\{0,1\right\}$ as the identifier of the divided image patches. The initial values of the elements of P are set as 0.4:**while** There are detected bounding boxes to be identified in the 1-th channel, i.e., j<n **do**5: Compute the starting indexes $s_{x}$ and $s_{y}$ for the *j*-th image patch, i.e., $s_{x}=ceil\left(x_{j}/33\right)$ and $s_{y}=ceil\left(y_{j}/33\right)$;6: Compute the ending indexes $e_{x}$ and $e_{y}$ for the *j*-th image patch, i.e., $e_{x}=ceil\left(\left(x_{j}+w_{j}\right)/33\right)$ and $e_{y}=ceil\left(\left(y_{j}+h_{j}\right)/33\right)$;7: **while** $s\geq s_{x}$ and $s\leq e_{x}$ **do**8:  **while** $t\geq s_{y}$ and $t\leq e_{y}$ **do**9:   $p_{st}=1$;10:   t=t+1;11:  **end while**12:  s=s+1;13: **end while**14: j = j+1;15:
**end while**
16:**while** There are channels to be sampled, i.e., i≥c **do**17: **while** s<wp **do**18:  **while** t<hp **do**19:   Denote the pixels of the image patch identified with $p_{st}$ in the *i*-th channel as $x_{ist}$;20:   **if** $p_{st}=1$ **then**
21:    Sample the image patch $x_{ist}$ with $\Phi_{h}$, i.e., $y_{ihst}=\Phi_{h}x_{ist}$;22:   **else**23:    Sample the image patch $x_{ist}$ with $\Phi_{l}$, i.e., $y_{ilst}=\Phi_{l}x_{ist}$;24:   **end if**25:   t=t+1;26:  **end while**27:  s=s+1;28: **end while**29: Concatenate all CS measurements sampled with $\Phi_{h}$ and $\Phi_{l}$ respectively, represent the values with 16-bit half-precision floats, and then obtain the half-precision CS measurement $y_{i}={\left[y_{ih}\right]}_{\mathrm{FP}16}\cup {\left[y_{il}\right]}_{\mathrm{FP}16}$ for the *i*-th channel;30: i=i+1;31:
**end while**
32:Combine $y_{i}$ sampled with multiple MRs in each channel and then obtain the final half-precision CS measurement y.


In Algorithm 1, the ceil() function returns the smallest integer that is not less than the given value. Zero padding is used to keep the CS sampled image patches constant in each channel, when the acquired images are divided into image patches of size 33×33. After obtaining the results of half-precision CS measurement with Algorithm 1, they are transmitted to high-performance servers for reconstructing CS images with the DNN-based CS method.

### 5.2. DNN-Based CS Reconstruction with Multiple MRs

As indicated in Figure 1, there are multiple CS reconstruction networks used in MRCS, and each one of them is responsible for recovering image patches sampled with the corresponding MRs. The CS reconstruction networks contain linear mapping layers and residual sub-networks [39]. Each linear mapping layer consists of one fully connected layer with 1089 neurons, and its 1089-dimension outputs are reshaped into 33×33 preliminary reconstructed image patches. Each residual sub-network contains four residual learning blocks and each block contains three convolutional layers.

The received half-precision CS measurements are first converted into the 32-bit single-precision ones. Second, the CS reconstruction networks take $y_{ih}$ and $y_{il}$ as the inputs respectively, and the linear mapping layers generate preliminary reconstructed image patches. Third, the residual sub-networks take these preliminary outputs as the inputs, and infer the reconstructed patches with the measurement rates $MR_{h}$ and $MR_{l}$ respectively. Finally, the reconstructed patches compose an intermediate reconstructed image for each channel. The reconstructed images in the different channels are then merged into a natural image as the final output of MRCS.

## 6. Experiment Approaches

### 6.1. Implementation Approaches

MYOLO3 is implemented with the Darknet neural network framework [47], while MRCS is implemented with Caffe [48] for a range of MRs {0.01,0.04,0.10,0.40} respectively [39]. The training and test approaches for MYOLO3 and CS with multiple MRs are described as follows.

#### 6.1.1. Training and Test for MYOLO3

PASCAL VOC [49] is a standard dataset for building and evaluating models for the object detection task. The combination of the PASCAL VOC 2007 and 2012 trainval dataset named VOC2007+2012 is used as the training dataset of the proposed MYOLO3. It contains 20 classes with 16,551 training images in the dataset. The dataset PASCAL VOC Test 2007 contains 4952 test images, and is used as the test dataset to evaluate the performance of MYOLO3.

MYOLO3 is trained on the VOC2007+2012 dataset for 200 epochs with a starting learning rate of $10^{-3}$. It uses a weight decay of 0.005, a momentum of 0.9 and data augmentation tricks such as random crops, hue, saturation an exposure shifts and multi-scale training.

#### 6.1.2. Training and Test for CS with multiple MRs

MRCS uses the training dataset that includes 91 images [37] to train the CS reconstruction model, which is used to recover natural images in the third stage of MRCS. To construct a scaled space, the original training images are resized to three scales, i.e., 0.75, 1, and 1.5. The weights of the linear mapping layers are initialized using Gaussian distribution with the standard variance of 0.01. Meanwhile, the weights of the convolutional layers in MRCS are initialized using Gaussian distribution with the standard variance of 0.001.

There are only 2171 test images that contain objects such as persons, bicycles, and cars in the dataset PASCAL VOC Test 2007. The object classes (e.g., persons, bicycles, and cars) are crucial in real scenarios such as surveillance and person retrieval [50]. Therefore, a new test dataset is created using these 2171 images for evaluating the performance of CS sampling and reconstruction with MRCS. This newly created test dataset is named VOC-pbc.

### 6.2. Evaluation Metrics

The evaluation of the proposed MRCS contains two parts. The one part is the performance of the proposed MYOLO3, which is measured with metrics shown in Section 6.2.1. The other part is the performance of CS sampling and reconstructed with multiple MRs, which is evaluated with metrics shown in Section 6.2.2.

#### 6.2.1. Metrics for MYOLO3

The proposed object detector MYOLO3 is measured with the following metrics. First, the computation cost is evaluated with the widely used indicator MAdds, which is the number of multiply-add operations in a model. Second, the model size is calculated with the number of parameters in the MYOLO3 model. Finally, the accuracy of MYOLO3 is tested on the popular object detection dataset PASCAL VOC Test 2007.

#### 6.2.2. Metrics for CS with Multiple MRs

Four evaluation metrics are adopted to measure the performance of CS sampling and reconstructed with the proposed MRCS. First, reconstruction quality is measured by the mean Peak Signal-to-Noise Ratio (mPSNR) on the VOC-pbc dataset with MRCS. Second, Average Precision (AP) is used to evaluate the usefulness of reconstructed images in terms of object detection. The 416×416 sized MYOLO3 model is employed to evaluate AP on the revised PASCAL VOC Test 2007 dataset, named VOC-revise dataset. The VOC-revise dataset is created by replacing images in the original PASCAL VOC Test 2007 dataset with the corresponding reconstructed images from the reconstructed VOC-pbc dataset with multiple MRs. Third, to evaluate the reduction in storage space, the mean Compression Ratios (mCR) for the reconstructed images are calculated by the size of the uncompressed RGB images against the size of the corresponding CS measurements during CS sampling with MRCS. Finally, to measure the reduction in network bandwidth for real-time transmission of CS measurements, the size of required network bandwidth (Bwidth) for real surveillance applications (assuming 25 images are sampled per second) is calculated with the size of CS measurements sampled with MRCS.

## 7. Evaluation Results

The performance of the proposed MYOLO3 is compared in Table 2. Meanwhile, the performance of CS sampling and reconstruction with the proposed MRCS is illustrated in Table 3, where AP^0.5^ denotes that the accuracy for a class of objects predicted with MYOLO3 on the VOC-revise test dataset when the value of Intersection over Union (IoU) is 0.5. It is crucial for image CS sampling in real scenarios to improve the quality of image regions containing objects, such that $MR_{h}\geq MR_{l}$ in the experimentation. $MR_{h}=MR_{l}$ is a special case wherein MRCS only uses one MR in CS sampling and reconstruction. $MR_{h}=1.00$ means that the image patches are directly extracted from the original image instead of using CS sampling.

### 7.1. Comparison with Other Object Detectors

To evaluate the performance of the proposed real-time object detector MYOLO3, experiments are performed with four state-of-the-art real-time object detectors, including Tiny-YOLOv2 [23], Tiny-YOLOv3 [24], MobileNet+SSD [26] and PeleeNet [51]. These models are trained on the combined VOC2007+2012 trainval dataset, and are tested on the PASCAL VOC 2007 test dataset with mAP when the value of IoU is 0.5.

As shown in Table 2, the proposed MYOLO3 achieves the highest mAP of 74.0 on the original VOC 2007 test dataset, with APs of three key object classes (i.e., person, bicycle, and car) at 81.4%, 84.0% and 85.2% respectively. It significantly improves the accuracy of one-stage object detectors, e.g., the accuracy increases by 6.5% and 3.6% compared to the state-of-the-art one-stage detectors MobileNet+SSD and PeleeNet respectively. MYOLO3 is the smallest model of size 4.80 million parameters, which is only approximately 1/4 of the size of Tiny-YOLOv2. Moreover, MYOLO3 has a lower computational cost than Tiny-YOLOv2 [23] and Tiny-YOLOv3 [24]. This is because that depthwise separable convolutions and bottleneck residual blocks used in MYOLO3 effectively reduce the computation cost by replacing standard convolutions, and the reduction of computation cost is discussed in Section 4.1 and Section 4.2. Unfortunately, MYOLO3 contains more layers than MobileNet+SSD [26] and PeleeNet [51] such that it leads to more computation cost. However, MYOLO3 achieves higher mAP than MobileNet+SSD and PeleeNet on the VOC dataset. Thus, it is more feasible to be deployed on devices with limited memory, such as intelligent surveillance drones and cameras, etc. Additionally, it is faster in terms of loading and updating from remote servers. Compared to the small version of YOLO model Tiny-YOLOv3, the accuracy of MYOLO3 increases by 16.1% while the computation cost of the model decreases by 764 million multiply-add operations.

Thus, compared with the other four state-of-the-art real-time detectors, the accuracy of MYOLO3 surpasses them by a large margin on the VOC detection task. Moreover, MYOLO3 is the smallest model architecture by further reducing parameters of the network. It achieves the trade-offs between model size, accuracy, and computation cost, such that it is more suitable for the requirements of mobile and embedded computer vision applications. In particular, it is more suitable for real-time object detection tasks in CS sampling with multiple MRs on resource-limited terminal surveillance devices.

### 7.2. Performance of CS Sampling and Reconstruction with Multiple MRs

The results of MRCS for CS sampling on the VOC-pbc dataset and corresponding CS reconstruction with multiple MRs are shown in Table 3. First, for CS sampling with one single measurement rate, both the quality of CS reconstructed images and the accuracy of object detection on them are improved when MRs are increased. However, this also significantly increases the quantity of CS measurement data. For instance, the size of CS measurements increases 27.26 times on average when $MR_{h}$ and $MR_{l}$ grow from 0.01 to 0.25. For reconstructed images when $MR_{h}$ and $MR_{l}$ are 0.01, they are less useful for applications related to object detection in real scenarios. The reason for this is that the AP is too low compared to the corresponding AP achieved on the original images. They only obtain 6.9%, 4.6% and 4.9% APs for the predicted persons, bicycles, and cars on the CS reconstructed images, respectively. Nevertheless, the corresponding original images obtain 81.4%, 84.0% and 85.2% APs, respectively.

Second, MRCS significantly reduces the size of CS measurements by sampling with multiple different MRs, such that it effectively reduces required network bandwidth and storage space to transmit and storage the CS measurements. The mCR significantly grows when $MR_{l}$ decrease, e.g., mCR increases by 61.89% for $MR_{h}=0.10$ when $MR_{l}$ drops to 0.04 from 0.10. This suggests that MRCS obtains a reduction in the size of CS measurements by sampling key regions with a higher measurement rate while the rest regions are sampled with a lower measurement rate. The reason for this is that the key region generally only makes up a small percentage of the entire image. Accordingly, the required transmission bandwidth is also rapidly declined, e.g., the required bandwidth reduces by 54.75% for real-time transmission of sampled CS measurements when $MR_{l}$ drops to 0.01 from 0.25 and $MR_{h}=0.25$.

Third, MRCS improves mPSNR and AP on the reconstructed images when the corresponding image patches containing pixels of objects use higher measurement rate than other image patches. For instance, the mPSNR and AP when $MR_{h}=0.10$ and $MR_{l}=0.01$ are higher than the ones when $MR_{h}$ and $MR_{l}$ are 0.01. For the same $MR_{l}$ in MRCS, mPSNR slightly increases with the growth of $MR_{h}$, while AP appreciably increases. For instance, the mPSNR is 19.45dB when $MR_{l}=0.01$ and $MR_{h}=0.04$, and the mPSNR only increases by 9.10% when $MR_{h}$ increases to 0.04 from 0.25. Nevertheless, the APs for the predicted persons, bicycles, and cars increase by 64.50%, 177.05%, 89.76% respectively. On the other hand, for the same $MR_{h}$ in MRCS, mPSNR and AP slightly decrease with the drop of $MR_{l}$, while mCR is significantly improved. For instance, when $MR_{h}=0.25$ and $MR_{l}$ drops to 0.01 from 0.10, the mCR increases by 125.87% while the mPSNR and AP only decrease by 13.77% and 7.25% on average, respectively.

Finally, reconstructed images with $MR_{h}=1.00$ get slightly lower APs than the original images. However, the mCR is gradually improved with a decrease in $MR_{l}$. For instance, the mCR for reconstructed images when $MR_{l}=0.01$ is 3.23 times larger than the mCR for the reconstructed images when $MR_{l}=0.25$.

Conceptually, a lower $MR_{l}$ and a higher $MR_{h}$ is useful for CS sampling and reconstruction in MRCS (e.g., $MR_{h}=0.25$ and $MR_{l}=0.01$), since it significantly reduces required network bandwidth and storage space while mPSNR and AP decrease slightly.

### 7.3. Performance of Half-Precision CS Measurements

MRCS obtains nearly identical performance for CS reconstruction in terms of mPSNR and AP when it transmits and stores CS measurements with single-precision floats and half-precision floats. For instance, for CS measurements represented with single-precision floats and half-precision floats when $MR_{h}=0.25$ and $MR_{l}=0.10$, MRCS obtains the same mCR of 23.19 and the approximate APs of 72.3%, 73.3% and 74.0% for the class of persons, bicycles, and cars, respectively. Notably, the mCRs of half-precision CS measurements are approximately one time larger than the mCRs of the corresponding single-precision ones. Moreover, the size of the required transmission bandwidth for half-precision CS measurements is only one half of the size of the corresponding single-precision ones. For instance, for $MR_{h}=0.25$ and $MR_{l}=0.10$, the mCR and required bandwidth of the half-precision CS measurements are 2.87 and 4.69Mb/s while the mCR of single-precision ones are 1.43 and 9.38Mb/s, respectively.

Thus, the size of CS measurements is further reduced by representing them with half-precision floats while preserving the usability of CS measurements in terms of both the reconstruction quality and the usability for object detection. This means that the storage and transmission of CS measurements with half-precision floats are suitable over the single-precision ones for further reducing the required transmission bandwidth and storage space for image CS sampling and reconstruction, especially for object detection applications in real scenarios with the limited network bandwidth and storage space.

## 8. Conclusions

The proposed MRCS is a low-cost image CS approach with multiple MRs and half-precision representation of CS measurements. It involves object detection with the proposed real-time object detector MYOLO3, CS sampling, and reconstruction with multiple MRs. It reduces the size of CS measurements in two ways: (1) The key regions are sampled with higher MRs while other regions are sampled with lower MRs. (2) Sampled CS measurements are represented with half-precision floats instead of single-precision floats during transmission and storage. The extensive experiments indicate that MRCS significantly reduces the required transmission bandwidth and storage space, while preserving the excellent quality of CS reconstruction and the usefulness of reconstructed images for object detection. Therefore, it is more suitable than existing CS methods for object detection-based applications such as the sampling and transmission of images/videos in large-scale distributed surveillance systems. Moreover, our proposed framework can be readily extended to the CS reconstruction of videos.

## Figures and Tables

**Table 1:** The architecture of MYOLO3. Each line describes a sequence of 1 or more identical layers, repeated *n* times. All layers in the same line have the same number $C_{out}$ of output channels. *s* denotes the stride used by each layer. *t* denotes the expansion factor used to resize input feature maps described by the height (*H*), width (*W*) and number of input channels ($C_{in}$), i.e., $H\times W\times C_{in}$. The number of detected object classes is denoted by cls.

Layers	Operation Type	Input	Filter Size	*s*	*t*	*c_out_*	*n*
1	Convolution	416 × 416 × 3	3 × 3	2	-	32	1
2–4	Bottleneck residual block	208 × 208 × 32	-	1	1	16	1
5–6	Bottleneck residual block	208 × 208 × 16	-	2	6	24	1
8–10	Bottleneck residual block	104 × 104 × 24	-	1	6	24	1
11–13	Bottleneck residual block	104 × 104 × 24	-	2	6	32	1
14–19	Bottleneck residual block	52 × 52 × 32	-	1	6	32	2
20–22	Bottleneck residual block	52 × 52 × 32	-	2	6	64	1
23–31	Bottleneck residual block	26 × 26 × 64	-	1	6	64	3
32–34	Bottleneck residual block	26 × 26 × 64	-	1	6	96	1
35–40	Bottleneck residual block	26 × 26 × 96	-	1	6	96	2
41–43	Bottleneck residual block	26 × 26 × 96	-	2	6	160	1
44–49	Bottleneck residual block	13 × 13 × 160	-	1	6	160	2
50–52	Bottleneck residual block	13 × 13 × 160	-	1	6	320	1
53	Convolution	13 × 13 × 320	1 × 1	1	-	1280	1
54	Convolution	13 × 13 × 1280	1 × 1	1	-	512	1
55–62	Depthwise separable convolution	13 × 13 × 512	-	1	-	512	4
63	Convolution	13 × 13 × 512	1 × 1	1	-	75 (cls = 20)	1
64	YOLO	-	-	-	-	-	1
65	Route	60	-	-	-	-	1
66	Convolution	13 × 13 × 512	1 × 1	1	-	256	1
67	Nearest neighbor upsampling	-	-	2	-	-	1
68	Route	67, 40	-	-	-	-	1
69	Convolution	26 × 26 × 352	1 × 1	1	-	256	1
70–77	Depthwise separable convolution	26 × 26 × 256	-	1	-	256	4
78	Convolution	26 × 26 × 256	1 × 1	1	-	75 (cls = 20)	1
79	YOLO	-	-	-	-	-	1
80	Route	75	-	-	-	-	1
81	Convolution	26 × 26 × 256	1 × 1	1	-	128	1
82	Nearest neighbor upsampling	-	-	2	-	-	1
83	Route	82, 19	-	-	-	-	1
84	Convolution	52 × 52 × 160	1 × 1	1	-	128	1
85–92	Depthwise separable convolution	52 × 52 × 128	-	1	-	128	4
93	Convolution	52 × 52 × 128	1 × 1	1	-	75 (cls = 20)	1
94	YOLO	-	-	-	-	-	1

**Table 2:** Performance comparison of real-time object detectors on PASCAL VOC 2007. M denotes million, mAP^0.5^ denotes that the accuracy predicted on the original PASCAL VOC 2007 test dataset when the value of IoU is 0.5.

Model	Input Size (Height × Width)	Computation Cost (MAdds)	Model Size (Parameters)	mAP^0.5^ (%)
Tiny-YOLOv2 [23]	416 × 416	3490 M	15.86 M	57.1
Tiny-YOLOv3 [24]	416 × 416	2742 M	8.72 M	58.4
MobileNet+SSD [26]	300 × 300	1150 M	5.77 M	68.0
PeleeNet [51]	304 × 304	1210 M	5.43 M	70.9
**MYOLO3 (ours)**	416 × 416	1978 M	4.80 M	74.0

**Table 3:** Performance of MRCS for CS sampling and reconstruction with multiple MRs. Bwidth denotes the size of required network bandwidth for real-time transmitting CS measurements generated when MRCS samples 25 images per second. AP^0.5^ denotes the accuracy predicted for one certain class on the VOC-revise dataset when the value of IoU is 0.5.

*MR_h_/MR_l_*	Single-Precision CS Reconstruction						Half-Precision CS Reconstruction					
	mCR	Bwidth (Mb/s)	mPSNR (dB)	AP^0.5^ (%)			mCR	Bwidth (Mb/s)	mPSNR (dB)	AP^0.5^ (%)		
				Person	Bicycle	Car				Person	Bicycle	Car
0.25/0.25	0.89	14.21	26.11	74.5	74.6	77.3	1.78	7.11	26.11	74.5	74.6	77.3
0.10/0.10	2.23	5.69	23.20	63.1	58.4	60.8	4.45	2.85	23.20	63.1	58.1	60.9
0.04/0.04	5.64	2.26	20.84	40.9	24.6	33.8	11.28	1.12	20.84	41.0	24.8	33.7
0.01/0.01	24.26	0.53	18.14	6.9	4.6	4.9	48.53	0.26	18.14	6.9	4.6	5.0
0.25/0.10	1.43	9.38	24.61	72.3	73.4	74.0	2.87	4.69	24.61	72.3	73.3	73.9
0.25/0.04	2.12	7.41	23.19	69.5	70.5	71.2	4.24	3.71	23.19	69.5	71.1	71.1
0.25/0.01	3.23	6.43	21.22	65.8	67.6	70.4	6.45	3.22	21.22	65.7	70.4	70.4
0.10/0.04	3.61	3.74	22.04	60.4	55.0	57.4	7.21	1.87	22.04	60.6	57.4	57.4
0.10/0.01	6.35	2.76	20.37	58.5	55.3	60.1	12.67	1.38	20.38	58.5	60.2	60.2
0.04/0.01	11.51	1.27	19.45	40.0	24.4	37.1	22.92	0.64	19.45	40.0	37.1	37.1
1.00/0.25	0.89	14.22	31.12	79.1	81.6	82.5	1.29	10.18	31.12	79.2	81.6	82.4
1.00/0.10	1.44	9.38	28.07	76.5	80.0	79.8	1.94	7.76	28.07	76.5	79.9	79.8
1.00/0.04	2.12	7.42	25.68	73.6	76.9	77.6	2.68	6.78	25.68	73.6	76.9	77.6
1.00/0.01	3.23	6.44	22.72	69.4	73.0	75.4	3.66	6.29	22.72	69.4	73.0	75.4
